# Glucagon-Like Peptide-1 agonists in perioperative medicine: to suspend or not to suspend, that is the question

**DOI:** 10.1016/j.bjane.2024.844538

**Published:** 2024-06-27

**Authors:** Florentino Fernandes Mendes, Lorena Ibiapina M. Carvalho, Maristela Bueno Lopes

**Affiliations:** a Universidade Federal de Ciências da Saúde de Porto Alegre (UFCSPA), Departamento de Clínica Cirúrgica, Programa de Pós-Graduação em Tecnologia da Informação e Gestão em Saúde, Porto Alegre, RS, Brazil; b Hospital Prontomed, Teresina, PI, Brazil; c Hospital Unimed Primavera, Departamento de Anestesiologia, Teresina, PI, Brazil; d Hospital São Marcelino Champagnat, Curitiba, PR, Brazil; e Pontifícia Universidade Católica do Paraná, Departamento de Anestesiologia, Curitiba, PR, Brazil

**“Although it is desirable that there be no solid matter in the stomach when chloroform is administered, it will be very salutary to give a cup of tea or beef broth about two hours before.”**

Sir Joseph Lister, 1883 British surgeon

In 1946, Mendelson described 66 cases of gastric content aspiration among 44,016 pregnant patients who underwent general anesthesia with ether and oxygen via facial mask for vaginal or cesarean delivery. He reported an incidence of pulmonary aspiration of 0.15%. Two patients died due to complete airway obstruction by solid residues. Mendelson further demonstrated that chloridric acid (or liquid vomit) caused immediate cyanosis, respiratory distress, and radiographic alterations when instilled into the lungs of anesthetized rabbits. These findings closely resembled those observed in patients who had experienced pulmonary aspiration. According to Mendelson, chemical pneumonitis following the aspiration of gastric contents was more likely to occur at a pH below 2.5. It was then recommended to withhold oral nutrition during the labor period, with no distinction between clear liquids and solids.^1^

Based on unpublished data involving the instillation of acidic content into the trachea of Rhesus monkeys, Roberts suggested that 0.4 mL.kg^-1^ was the maximal volume of aspirated acid that would not cause significant pulmonary alterations. This volume was estimated to be equivalent to 25 mL in an adult female human.^2^

The formal definition of patient at risk of aspiration was therefore postulated as a minimal amount of 25 mL of gastric juice with a pH below 2.5. In the subsequent decades, this threshold became known as the critical gastric volume and supported the practice of *nil per os* (NPO) after midnight for elective surgical procedures.

The American Society of Anesthesiologists (ASA) guidelines recommend a minimum fasting period for healthy patients as follows: 2 hours for clear liquids, 4 hours for human breast milk, 6 hours for non-human milk, formula milk, and light meals, and 8 hours for fried or fatty solids or meat.^3^ However, actual fasting times frequently exceed these limits due to factors such as delayed surgery schedules, cultural influences, and misleading preoperative guidance.^4^ Given the substantial evidence regarding the adverse effects of prolonged fasting during the perioperative period,^3^ abbreviated fasting protocols with carbohydrate drinks should be the standard practice, not the exception.

Gastric emptying of clear liquids is primarily influenced by the volume and energy density of the ingested fluid. Higher volumes and liquids with lower energy density are emptied more rapidly, whereas higher caloric intake is associated with slower emptying rate. Carbohydrates exit the stomach faster than proteins, and lipids have the slowest rate of emptying. In volunteers, the complete emptying of the stomach after consuming 500 mL of water occurs in approximately 20 minutes. Additionally, gastric emptying is not affected by age or the ingestion of liquid carbohydrates.^1^

## Risk factors for pulmonary aspiration

Recent studies have defined the normal gastric fluid volume as up to 1.5 mL.kg^-1^. The value represents the upper threshold for baseline gastric secretions. Based on this standard, it has been demonstrated that 31% of gynecological patients and 48% of diabetic patients may have increased gastric fluid volume despite fasting.^5^^,^^6^

Similar to observations in Mendelson's risk group, perioperative pulmonary aspiration during elective surgeries in both adults and children is rare. Consequently, prospective studies investigating pulmonary aspiration as a primary outcome face the challenge of requiring an impractically large sample size to achieve sufficient statistical power.^4^

The aspiration of clear fluids rarely has clinical significance, in contrast to the aspiration of solid materials, which leads to severe complications. Additionally, it is crucial to understand that the risks of pulmonary aspiration go beyond gastric emptying time. In fact, aspiration pneumonia, or chemical pneumonia, is the final stage of a sequence of events that includes: (1) the presence of a significant volume of gastric contents; (2) delayed or inadequate gastric emptying; (3) dysfunction of the lower esophageal sphincter; (4) regurgitation of gastric residue; (5) a sufficient volume of gastric content reaching the bronchus; and (6) pulmonary injury.^1^

Therefore, gastric volume alone cannot reliably indicate the risk of pulmonary aspiration or the occurrence of chemical pneumonia.

## Glucagon-Like Peptide-1 Receptor Agonists (GLP1-RA)

Endogenous GLP-1 is an incretin hormone derived from the intestine that lowers blood glucose levels by stimulating insulin production and secretion from pancreatic beta cells, while reducing glucagon secretion from alpha cells. Additionally, GLP-1 inhibits gastric emptying, suppresses appetite, and reduces food intake, all of which contribute to lowering blood glucose levels. Notably, the pancreatic effects of GLP-1 occur exclusively during hyperglycemia, thereby minimizing the risk of hypoglycemia. Endogenous GLP-1 has a very short half-life of several minutes and is rapidly degraded by dipeptidyl peptidase-4 (DPP-4).^7^

GLP-1 receptors are widely distributed in extra-pancreatic tissues, including the heart, vascular smooth muscle, lungs, kidneys, gastrointestinal tract, hypothalamus, and vagus nerve. This broad distribution suggests potential systemic effects related to this class of drugs.^8^

Short-acting agents like exenatide and lixisenatide delay gastric emptying and reduce postprandial hyperglycemia. Long-acting agents such as liraglutide, extended-release exenatide, dulaglutide, and semaglutide enhance insulin secretion and inhibit glucagon release, thus regulating blood glucose levels.^7^

When the GLP-1 receptor is activated by an agonist, it results in reduced blood glucose levels by enhancing insulin secretion and inhibiting glucagon release. At higher doses, it induces appetite suppression and weight loss, which are achieved through a combination of central effects on the hypothalamus and delayed gastric emptying. From a public health standpoint, GLP-1 receptor agonists hold significant potential, especially given the widespread challenges of obesity and type 2 diabetes globally.^9^

Recent clinical studies have identified an increased proportion of gastric content retention in patients taking GLP1-RAs, especially semaglutide.^10^, ^11^, ^12^, ^13^, ^14^ Indeed, gastric retention is a prevalent issue among all GLP1-RAs and warrants further investigation.^15^ The risk of aspiration during general anesthesia or sedation, although considered low, remains incompletely understood in this scenario. This underscores the need for caution in patients who have recently initiated GLP1-RA therapy.^16^

In fact, the recent introduction of these medications has raised concerns regarding patient safety during general anesthesia or sedation and has prompted the search for precise guidelines regarding the best course of action. Although there are some formal recommendations on the management of GLP-1 RAs, there is still no consensus on the ideal suspension period among different Anesthesiology societies.^17^, ^18^, ^19^, ^20^ The suggested suspension periods vary widely: from continuing the medication use unchanged,^19^ to suspending short-acting GLP-1 RAs on the day of surgery and to suspending long-acting GLP-1 RAs for 7 days,^17^ to suspending semaglutide for 21 days,^20^ or basing suspension on three half-lives of the medication.^18^^,^^21^ It is important to note that there is no clear distinction regarding dosage, duration of medication use, type of diet, age, risk factors, or initial indication for use (e.g., diabetes, obesity) among the available guidelines.

According to the ASA consensus guideline, GLP-1 RAs should be discontinued on the day of the procedure/surgery for patients on daily dosing, and one week before surgery for those on weekly dosing. On the day of the procedure, if patients have not discontinued the medication, surgery should be postponed and risks discussed, especially if there are gastrointestinal symptoms (GI). In the absence of GI symptoms, the decision should be guided by an assessment using gastric ultrasound.^17^

Additionally, these recommendations were based on retrospective studies and some case reports^7^, ^12^, ^22^ that showed an association between residual gastric contents (solid and liquid) and the use of GLP-1 RAs in patients undergoing gastroduodenal endoscopy with adequate fasting times.^13^^,^^14^

Interestingly, a cross-sectional study using gastric ultrasound in fasting patients who discontinued GLP-1 RA for 7 days before elective procedures *versus* controls found a 30.5% higher prevalence of residual gastric content in the GLP-1 RA group (adjusted prevalence ratio of 2.48).^23^ These findings highlight the need for prospective randomized research to better understand the issue.

On the other hand, the use of GLP1-RA medications is anticipated to rise. Indeed, current research is investigating their potential benefits for several prevalent conditions, including metabolic dysfunction-associated steatotic liver disease, polycystic ovary syndrome, juvenile diabetes and obesity, among others.^24^ However, these medications are presently available only through patented commercial formulations, posing financial barriers to widespread usage. With the upcoming expiration of these patents, the resulting availability of generic formulations is expected to substantially enhance accessibility and usage.^25^

Cardiovascular outcome studies have confirmed that GLP1-RAs are safe and do not increase the risk of major adverse cardiovascular events (MACE). On the contrary, several studies have shown reduction in the risk of MACE associated with the use of GLP1-RAs compared to standard treatment. Major cardiovascular outcome studies have identified clear cardiovascular benefits, with reductions in the rates of myocardial infarction, stroke, and revascularization procedures.^26^

The growing body of evidence features the need for detailed research into these medications and their effects on anesthetic management. Continuing these medications could offer more benefits than risks, and further research and clinical trials are necessary to corroborate this hypothesis.^27^^,^^28^

Indeed, GLP1-RAs have demonstrated various positive perioperative effects. As these medications have proven to be effective in the treatment of Type 2 Diabetes Mellitus, their discontinuation may raise concerns regarding impaired blood glucose control, once hyperglycemia increases perioperative complications and mortality.^29^

It is reasonable to assume that appropriate preoperative fasting is practiced for all elective surgeries in which temporary cessation of medication has been considered. Consequently, postoperative glucose dysregulation may be linked to the absence of endocrine effects from GLP1-RA in patients who temporarily discontinue these medications. While this is an important consideration, the clinical significance of the finding still lacks literature to counterbalance the low aspiration risk possibly associated with GLP1-RA.^27^^,^^28^

An important consideration for these drugs is the previously documented rapid-onset tachyphylaxis. The effects on vagal inhibition, such as nausea and gastroparesis, can be significantly reduced by the second dose of a GLP1-RA, stabilizing after 5 to 8 weeks.^10^^,^^15^ While tachyphylaxis is a known phenomenon, further research is necessary to understand the factors influencing it and its impact on dose selection and surgical planning. Currently, it is advisable to avoid general anesthesia or sedation a short period after the initiation of these medications, if possible.^27^

Given these considerations, the assessment of gastric volume using ultrasonography is a valuable perioperative tool. Despite its limitations of representing an isolated measurement within a dynamic process, its use immediately before surgery can aid in decision-making and enhance safety in patients with delayed gastric emptying, such as those using GLP1-RA.^23^^,^^30^

Determining the optimal timing for discontinuing GLP1-RAs is critically important. To minimize perioperative risks and maintain glycemic control, research must pursue the most suitable interval for ceasing GLP1-RAs prior to surgery. It is essential to establish evidence-based guidelines, derived from well-designed clinical studies, concerning timing of discontinuation, dose range, duration of effect, their half-lives, and how they interact with specific patient variables. Addressing these issues will help to enhance perioperative treatment options for patients on GLP1-RAs, thereby improving patient safety and outcomes of medical procedures.^27^

## Final considerations

GLP1-RAs can delay gastric emptying, potentially increasing the risk of pulmonary aspiration. This could pose a significant hazard for surgical patients using these medications. Although these concerns are based primarily on case reports and limited retrospective studies, they emphasize the necessity for additional investigation and careful management.

Thus, the provided considerations attempt to balance the risk of a rare, but potentially fatal, aspiration event against the risks of weight (re)gain, loss of cardiovascular benefits, and poorer perioperative glycemic control. The need for high-quality scientific research is urgent, to better understand this issue, if it exists, and to develop appropriate solutions.

From Mendelson's observations to the present, our understanding of the risk of pulmonary aspiration related to anesthesia has significantly evolved. However, we still lack sufficient evidence to provide conclusive recommendations regarding the safety of discontinuing, or continuing, GLP1-RA in elective surgeries.

## Declaration of competing interest

The authors declare no conflicts of interest.
